# The beneficial use of ultramicronized palmitoylethanolamide as add-on therapy to Tapentadol in the treatment of low back pain: a pilot study comparing prospective and retrospective observational arms

**DOI:** 10.1186/s12871-017-0461-9

**Published:** 2017-12-19

**Authors:** Maria Beatrice Passavanti, Marco Fiore, Pasquale Sansone, Caterina Aurilio, Vincenzo Pota, Manlio Barbarisi, Daniela Fierro, Maria Caterina Pace

**Affiliations:** 1 Department of Women, Child and General and Specialized Surgery, University of Campania “Luigi Vanvitelli”, 80138 Naples, Italy; 2 Department of Medical, Surgical, Neurological, Metabolic and Aging Sciences, University of Campania “Luigi Vanvitelli”, Piazza L. Miraglia, 2, 80138 Naples, Italy

**Keywords:** Ultramicronized palmitoylethanolamide, Low back pain, Tapentadol, Add-on therapy, Pain medicine

## Abstract

**Background:**

This pilot study was designed to compare the efficacy of ultramicronized palmitoylethanolamide (um-PEA) as add-on therapy to tapentadol (TP) with TP therapy only in patients suffering from chronic low back pain (LBP).

**Methods:**

This pilot observational study consists in two arms: the prospective arm and the retrospective one. In the prospective arm patients consecutively selected received um-PEA as add-on therapy to TP for 6 months; in the retrospective arm patients were treated with TP only for 6 months. Pain intensity and neuropathic component were evaluated at baseline, during and after 6 months. The degree of disability and TP dosage assumption were evaluated at baseline and after 6 months.

**Results:**

Statistical analysis performed with generalized linear mixed model on 55 patients (30 in the prospective group and 25 in the retrospective group) demonstrated that um-PEA as add-on treatment to TP in patients with chronic LBP, in comparison to TP alone, led to a significantly higher reduction in pain intensity, in the neuropathic component, the degree of disability and TP dosage assumption. No serious side effects were observed.

**Conclusion:**

Overall, the present findings suggest that um-PEA may be an innovative therapeutic intervention as add-on therapy to TP for the management of chronic LBP with a neuropathic component, as well as to improve patient quality of life. Additionally, this combination treatment allowed a reduction in TP dose over time and did not show any serious side effects.

## Background

Low back pain (LBP) is one of the most common chronic pain conditions encountered in clinical practice worldwide, with a lifetime prevalence estimated to be >70% in industrialized countries [1]. LBP is considered chronic when it persists for 12 weeks or more and is frequently associated with comorbid conditions, especially depression, panic and anxiety disorders, and sleep disturbances. The complexity and heterogeneity of chronic LBP may involve both nociceptive and neuropathic pain mechanisms: the former from activation of nociceptors in response to tissue injury and/or inflammation and biomechanical stress, and the latter from injury or disease that directly affect nerve roots innervating the spine and lower limbs, thereby resulting in pathological innervation of the damaged lumbar discs [2].

Today’s management of chronic LBP includes analgesics (paracetamol), non-steroidal anti-inflammatory drugs, antidepressants, anticonvulsants, opioids, and topical treatments [3], with oral agents recommended as first-line therapy. Analgesics and non-steroidal anti-inflammatory drugs target the nociceptive component of LBP without affecting neuropathic pain components, while opioids target both nociceptive and (to a lesser degree) neuropathic pain, and antidepressants target only the neuropathic component, although data concerning their efficacy is conflicting [2, 4]. Although relieving neuropathic pain to some extent, classical opioid analgesics suffer from frequent side effects, particularly at the gastrointestinal level that limit their long-term use [5]. Tapentadol (TP) is a centrally-acting analgesic with broad activity achieved by combining two established analgesic principles (μ-opioid receptor agonism and noradrenaline reuptake inhibition) in a single molecule, and offers a better balance between efficacy and tolerability than classical opioids [6].

Immune cells, such us mast cells and microglia, have an important role as pain modulators not just in inflamed tissues, but also in damaged peripheral nerves and in the central nervous system [7–9]. Degranulation of mast cells close to peripheral nerve terminals leads to nociceptor sensitization, a condition whereby nociceptor threshold activation decreases and neuronal cell excitability rises [10, 11]. Persistent peripheral sensitization can enhance responsiveness of spinal cord neurons, leading to microglia activation which contributes to central sensitization and the development of chronic pain [12, 13]. An innovative approach in the management of chronic pain diseases is represented by palmitoylethanolamide (PEA), a member of the N-acylethanolamine family, produced by most mammalian cells and which is particularly abundant in brain tissues [14]. PEA is involved in endogenous protective mechanisms activated by stimulation of inflammatory responses. PEA exerts its effects on cellular targets involved in the generation and maintenance of pain [15] by down-modulating mast cell activation and controlling microglial cell behaviors.

Ultramicronized-PEA (um-PEA) and micronized-PEA (m-PEA) have been used clinically in the treatment of various syndromes associated with chronic pain that are poorly responsive to standard therapies [16–20]. Importantly, m-PEA displayed pain-relieving properties in patients affected by lumbosciatalgia, mainly caused by nerve root compression [21]; m-PEA treatment in chronic lumbosciatalgia was accompanied by a significant reduction in non-steroidal anti-inflammatory drug use [22]. Um-PEA administered as add-on therapy with low doses of oxycodone led to a good pain control and excellent tolerability, suggesting that um-PEA may allow for a reduction of opioid dose and related side-effects [23]. Um-PEA may thus be a valid add-on therapy to minimize the risks of chronic opioid treatment in diseases associated with chronic pain of a neuropathic nature. Based on these observations, this pilot study was designed to compare the efficacy of um-PEA as add-on therapy to TP with TP therapy only in patients suffering from LBP.

## Methods

Patients affected by LBP were recruited in the Pain Therapy outpatient clinic at the University of Campania “Luigi Vanvitelli”. Inclusion criteria were: age ≥ 18 years; diagnosis of LBP; presence of neuropathic pain for at least 6 months; stability of painful symptoms for at least 3 months; pain intensity score ≥ 6 measured by visual analog scale (VAS) (in reference to the day before study start); DN4 (douleur neuropathique 4 questions) ≥4; presence of hyperalgesia and allodynia by pinprick test and brush test. The study was performed in compliance with the Good Clinical Practice guidelines and the principles of the Declaration of Helsinki of 1964 and its subsequent revisions; it followed STROBE guidelines for the reporting of observational studies and was communicated to the departmental Review Board.

This pilot observational study consists in two arms: a prospective arm and a retrospective arm, which have been compared to each other. In the prospective arm, data were collected from recruited patients in appropriate case report forms, while in the retrospective arm data from patients complying with inclusion criteria were collected from their clinical charts. All collected data were then transferred to a database.

All patients considered in this study were treated for 6 months; in the prospective group the patients, selected consecutively in the period between October 2014 and March 2015, received um-PEA microgranules 600 mg [Normast®, Epitech Group SpA, Saccolongo, Italy] twice daily as adjuvant to TP. Retrospective group patients were treated with TP only for 6 months between January 2013 and June 2014. The dose of TP could vary between 100 mg and 500 mg depending on patient needs, as established by normal clinical practice. Paracetamol (1000 mg) was habitually used as rescue drug in case of exacerbations of pain.

In all patients, the main outcomes were: i) pain intensity evaluated by the VAS. The VAS is a continuous scale comprised of a horizontal line, 10 cm (100 mm) in length, anchored by 2 verbal descriptors, one for each symptom (0 = no pain and 10 = the worst pain imaginable). ii) Neuropathic pain component detected by DN4 (Douleur Neuropathique 4). DN4 questionnaire included four questions consisting of both sensory descriptors and signs related to bedside sensory examination [24]. iii) Permanent functional disability evaluated with ODQ (Oswestry Disability Questionnaire) [25]. iv) Tolerability and monitoring of possible side effects registered on specifics forms, if found. All parameters were assessed at baseline (T0) and at the programmed follow-up after 3 weeks (T1), 12 weeks (T2) and 24 weeks (T3–end of treatment), as usual in our pain outpatient clinic.

The above parameters are evaluated as part of normal clinical practice in the pain therapy outpatient clinic at the University of Campania “Luigi Vanvitelli”, for afferent patients affected by chronic low back pain. Statistical analysis was performed using the generalized linear mixed model (GLMM). Variables such as gender and age were included in the model as covariates. All scores are given as mean ± standard error (S.E.) unless otherwise specified. Responder analysis was also performed. For this propose, minimal important difference (MID) was established as fixed effects, since percentage reductions depend on baseline values. In particular, a patient was considered a responder in case of: i) pain intensity reduction of at least 2, 3, or 4 points or by achieving a VAS score ≤ 3 (gold standard) [26]. ii) neuropathic pain component reduction of at least 1, or 2 points or by achieving a DN4 score < 4 (gold standard) [24]. iii) functional disability reduction of at least 10, 15 or 20 points on ODQ, where a 15-point reduction is the gold standard [27]. Scores are given as a percentage. A *p*-value of less than 0.05 was considered significant for both GLMM and responder analysis. All patients for both groups were evaluated at each time point.

## Results

**Fig. 1:**
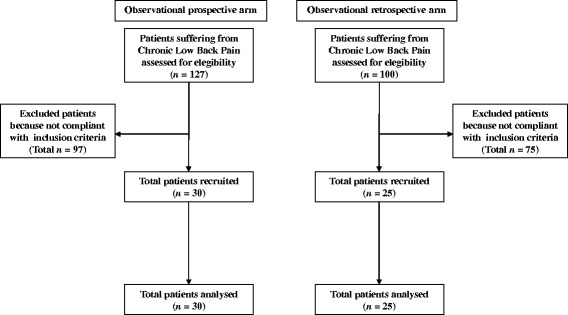
Patient flow chart

Fifty-five patients affected by chronic LBP were considered for this study; 30 patients (21 females, 9 males; mean age ± S.D. of 64.7 ± 4.12) were enrolled in the prospective group treated with um-PEA as add-on to TP (um-PEA-TP group); 25 patients (20 females, 5 males; mean age ± S.D. of 64.1 ± 4.79) were included in the retrospective group treated with TP only (TP group) (Fig. 1). The mean daily dose of TP administered was 196.0 mg in the um-PEA-TP group and 203.3 mg in the TP group; no significant differences were observed between groups at baseline. Patient anamnestic and demographic data are reported in Table 1.

**Table 1:** Patient demographic and medical information

	TP group	um-PEA-TP group
Age (mean ± S.D.)	64.1 ± 4.79	64.7 ± 4.12
Gender	5 (M) 20 (F)	9 (M) 21 (F)
Body weight (Kg -mean ± S.D.)	75.3 ± 13.7	77 ± 11.5
Height (cm -mean ± S.D.)	165 ± 5.5	162 ± 7.5
Caucasian (%)	100	100
Comorbidities (%):		
diabetes mellitus type II	40	40
hypertension	28	20
ischemic heart disease	20	13.3
dyslipidemia	12	0
morbid obesity	16	20
diverticular disease of the colon	8	3.3
chronic obstructive pulmonary disease	36	33.3
heart rhythm disturbances	4	3.3
osteoporosis	48	43.3
obstructive arterial disease	4	0
chronic venous insufficiency of the lower limbs	4	23.3*

*p < 0.05

**Fig. 2:**
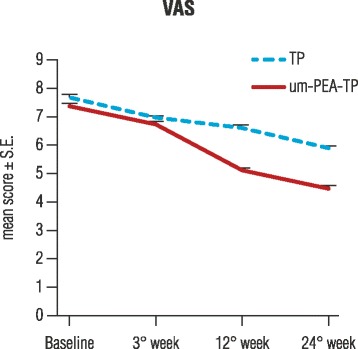
Pain intensity evaluation Visual analog scale (VAS) scores obtained at baseline, during and after 24 weeks in the group treated with um-PEA as add-on to tapentadol (um-PEA-TP group) or with tapentadol only (TP-group). Data are means ± S.E. Both groups showed a significant reduction in low back pain intensity over time (*p* < 0.0001). A further, significant difference (*p* < 0.0001) between the two groups was found in favor of the um-PEA-TP group. Statistical analysis were performed with GLMM.

Statistical analysis with GLMM demonstrated VAS mean pain intensity score to decrease significantly over time in both groups when considered separately (*p* < 0.0001). VAS values in the um-PEA–TP group decreased from 7.4 ± 0.08 (T0) to 4.5 ± 0.09 (T3), and in the control TP group from 7.7 ± 0.10 (T0) to 5.9 ± 0.09 (T3). The reduction in VAS mean score was statistically significant in the um-PEA–TP group compared to the TP group (*p* < 0.0001) (Fig. 2).

**Fig. 3:**
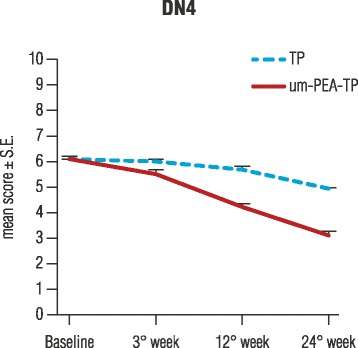
Neuropathic component evaluation. Douleur neuropathique 4 questions (DN4) score obtained at baseline, during and after 24 weeks in the group treated with um-PEA as add-on to tapentadol (um-PEA-TP group) or with tapentadol alone (TP group). Data are means ± S.E. The neuropathic component decreases significantly over time in both groups (*p* < 0.0001). A further, significant improvement over time was found in the um-PEA-TP group compared to the TP-only group (*p* < 0.0001). Statistical analysis were performed with GLMM

**Fig. 4:**
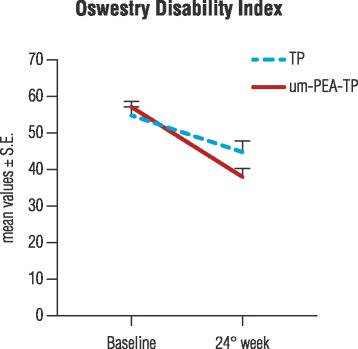
Low back pain/dysfunction evaluation. Oswestry Disability Index at baseline and after 24 weeks of treatment for the groups with um-PEA as add-on to tapentadol (um-PEA-TP) or with tapentadol alone (TP). Mean values ± S.E. The degree of disability decreased significantly between baseline and week 24 in both groups (*p* < 0.0001). There was a significantly higher reduction (*p* < 0.0012) in favor of the um-PEA-TP group compared to the TP-only group. Statistical comparisons were performed with GLMM

DN4 questionnaire scores showed that the neuropathic component significantly decreased over time in both groups (*p* < 0.0001), with a significantly higher reduction in favor of the prospective group (*p* < 0.0001) (Fig. 3). Patients in the um-PEA-TP group with a DN4 mean score of 6.1 ± 0.14 at baseline (T0), reached a mean score of 3.2 ± 0.13 (T3) at treatment end; patients in the TP group with a mean score of 6.1 ± 0.09 (T0) achieved a mean score of 5.0 ± 0.04 at T3. Furthermore, the ODQ questionnaire score evidenced a significant reduction between baseline and treatment end in both groups (*p* < 0.0001) (Fig. 4). The mean score for the um-PEA-TP group decreased from 56.9 ± 1.55 (T0) to 37.7 ± 2.38 (T3), while for the TP-only group it decreased from 54.6 ± 2.20 to 44.6 ± 3.02 showing a further significant difference in the um-PEA-TP group compared to the TP group (*p* < 0.0012).

**Fig. 5:**
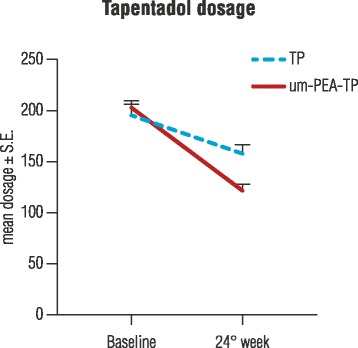
Doses of tapentadol taken over time. Tapentadol dosage taken at baseline and after 24 weeks for groups treated with um-PEA as add-on to tapentadol (um-PEA-TP group) or with tapentadol alone (TP group). Mean values ± S.E. Both groups reduced significantly their TP dosage between baseline and week 24 (*p* < 0.0001). There was a greater reduction (*p* < 0.0001) in favor of the um-PEA-TP group over the TP-only group. Statistical comparisons were performed with GLMM

TP dosage was significantly reduced in both groups between baseline and treatment end (*p* < 0.0001), with a greater reduction in favor of the um-PEA-TP group (*p* < 0.0001). In fact, patients in the um-PEA-TP group received a mean dose of 203.3 ± 6.75 mg at study start (T0) which decreased to 121.7 ± 6.20 mg after 6 months (T3). In contrast, patients in the TP group initially took a mean dose of 196.0 ± 10.38 mg (T0), which decreased to 158.0 ± 8.50 mg (T3) (Fig. 5).

Neither age nor gender influenced the results of the parameters considered. Paracetamol (1000 mg) was used as rescue medication in 10% of patients in um-PEA-TP group vs 12% of patients in the TP-only group. This study did not present missing data. No serious side effects were reported/observed, although episodes of diarrhea occurred in 15% of patients in the um-PEA-TP group. Percentages of responder patients for each established MID and gold standard are given in Table 2.

**Table 2:** Percent of responders in um-PEA TP and TP groups at different follow-up times

Parameters	Goals	Percent of responders in um-PEA TP group/TP group at different follow-up times					
		T1		T2		T3	
VAS	Reduction						
	≥2	3.3	0	90***	16	100***	68
	≥3	3.3	0	30**	0	63.3***	8
	≥4	0	0	0	0	20*	0
	VAS score^a^ ≤ 3	0	0	0	0	0	0
DN4	Reduction						
	≥1	46. 7**	16	93.3***	36	100.0	96
	≥2	6. 7	0	73.3***	4	86. 7***	20
	DN4 score^b^ < 4	3. 3	0	6. 7	0	76. 7***	0
ODQ	Reduction						
	≥10	–	–	–	–	86.7**	48
	≥15	–	–	–	–	70.0**	24
	≥20	–	–	–	–	53.3**	16

^a^Percentage of patients who achieved a VAS score ≤ 3

^b^Percentage of patients who achieved a DN4 score < 4

*p < 0.05

**p < 0.01

***p < 0.0001

## Discussion

Chronic LBP is a common chronic pain condition [28] often associated with a neuropathic pain component [2, 29]. The presence of the latter denotes a more severe condition, which is complex, clinically challenging to manage and requires a multimodal treatment approach [30–32]. The present pilot observational study shows that um-PEA as add-on treatment to TP in patients with chronic LBP, in comparison to TP alone, leads to a significantly higher reduction in pain intensity, in the neuropathic component, the degree of disability and in TP dosage assumption. All study patients had chronic LBP for at least 6 months with baseline VAS scores ≥6 for pain intensity and a score ≥ 4 on the DN4 questionnaire, the latter taken as an index of a neuropathic component. VAS and DN4 mean scores decreased significantly over time in the two study groups (*p* < 0.0001) showing a further significant reduction in favor of the um-PEA-TP group (p < 0.0001) (Figs. 1 and 2). These results confirm the efficacy of TP for the management of moderate-to-severe chronic pain [33] and show, for the first time, the effectiveness of a combination therapy with um-PEA and TP in the treatment of chronic LBP. Prior clinical studies showed the pain-relieving properties of um-PEA and m-PEA alone and as add-on treatment for chronic pain associated with different disease etiologies without [16–18, 21, 34] and with a neuropathic component [19, 20].

The improved effectiveness of this combination therapy in the management of chronic LBP may be attributed to different mechanisms of action for um-PEA and TP in pain processes. PEA acts on immune-derived non-neuronal cells, in particular microglia and mast cells, which are involved in pain signalling but not directly in the transmission of pain perception [12, 15]. There is some evidence in support of a “receptor mechanism” based on the capability of PEA to directly stimulate either an as-yet uncharacterized cannabinoid CB2-like receptor expressed on these immune cells, or the nuclear receptor peroxisome proliferator-activated receptor-alpha, which appears to mediate many of the anti-inflammatory effects of PEA. Another proposed mechanism is the so-called “entourage effect”, which postulates that PEA acts by enhancing the anti-inflammatory and anti-nociceptive effects exerted by anandamide, which is often produced together with PEA and activates cannabinoid CB1 and CB2 receptors or the transient receptor potential vanilloid receptor type 1 channel [35]. TP is a centrally-acting analgesic with a bimodal mechanism of action, that is, μ-opioid receptor agonism and noradrenaline reuptake inhibition [36]: agonism of μ-opioid receptors interferes with pre- and postsynaptic transmission of ascending spinal cord pain signals while activating descending inhibitory projections at the supraspinal level, and noradrenaline reuptake inhibition raises noradrenaline content in the synaptic cleft to enhance pain inhibition in the descending pathways [33]**.** By modulating the activation of non-neuronal cells that normally controls neuronal cell sensitization, PEA can effect TP action on neurons, thus promoting a major remission of chronic pain also with a neuropathic component.

Both the um-PEA-TP and TP groups received a similar mean dose of TP. After 6 months of treatment, reduction in pain intensity was associated with a reduced TP mean dosage in both groups (*p* < 0.0001). Importantly, the reduction of TP mean intake was significantly higher in the um-PEA-TP group (p < 0.0001) (Fig. 3). These results confirm findings in another clinical study where PEA treatment in chronic lumbosciatalgia was accompanied by a significant reduction in non-steroidal anti-inflammatory drug use [34]. Further, m-PEA is reported to render efficacious, in terms of analgesic activity, sub-active doses of oxycodone in patients with LBP [23].

The association of um-PEA and TP led to a significant reduction compared to TP alone in the degree of disability measured by the ODQ low back pain questionnaire (Fig. 4). Responder analysis showed that main MID evaluated such as: the pain intensity reduction of at least 3 points on the VAS score, achievement of a DN4 score lower than 4 and functional disability reduction of at least 20 points on ODQ, were achieved primarily in the um-PEA-TP group. All the other MID assessed were achieved in both groups over time but more rapidly and with a higher percentuage of responders in the um-PEA-TP group as compared to the TP group.

It is important to point out also that none of the patients showed serious adverse effects. In the um-PEA-TP group only 15% of patients presented some episodes of diarrhea which did not necessitate discontinuation of therapy. Several clinical studies already support the tolerability both of um-PEA [15] and TP [33].

There was no statistically significant group difference in the incidence of dylipidemia (*p* = 0.0510). As far as were are aware of, no study until now has evaluated this interaction in clinical practice. There was, however, a modest but significant association between chronic venous insufficiency and um-PEA-TP (*p* = 0.0429) (Table 1). Chronic venous insufficiency is often associated with venous skin ulceration and discomfort, pain and compromised quality of life [37].

Future studies are needed to evaluate a possible direct effect of PEA on chronic venous insufficiency.

Caveats of the present observational study include lack of blindness and small patient sizes, which increases the difficulty in estimating the size effect of combination treatment. Furthermore, a treatment period longer than 6 months would allow one to better appreciate effectiveness and tolerability of the drug combination. In spite of these limitations, this exploratory study provides encouraging findings on the potential benefits of PEA addition to TP in patients affected by chronic LBP, and should stimulate confirmatory trials with larger patient cohorts.

## Conclusions

Overall, our findings suggest that um-PEA may be an innovative therapeutic intervention as add-on therapy to TP for the management of chronic LBP with a neuropathic component, also to improve patient quality of life – at least for a treatment period of 6 months. Additionally, this combination treatment allowed a reduction in TP dose over time and did not show any serious side effects. Um-PEA could thus allow for the maintenance of low dosages of TP, thereby delaying the occurrence of serious side effects of this drug over longer periods. Further prospective studies with a double-blind design and larger patient numbers are needed to confirm our findings.

## Abbreviations

DN4: douleur neuropathique 4
GLMM: Generalized linear mixed model
LBP: low back pain
MID: Minimal important Difference
ODQ: Oswestry Disability Questionnaire
TP: tapentadol
um-PEA: Ultramicronized palmitoylethanolamide
VAS: visual analog scale
